# Add-on histamine receptor-3 antagonist for allergic rhinitis: a double blind randomized crossover trial using the environmental exposure unit

**DOI:** 10.1186/1710-1492-10-33

**Published:** 2014-07-03

**Authors:** Michelle L North, Terry J Walker, Lisa M Steacy, Barnaby G Hobsbawn, Richard J Allan, Frances Hackman, Xiaoqun Sun, Andrew G Day, Anne K Ellis

**Affiliations:** 1 Department of Biomedical and Molecular Sciences, Queen’s University, Kingston, Ontario, Canada; 2 Allergy Research Unit, Kingston General Hospital, Kingston, Ontario, Canada; 3 Pfizer Ltd., Sandwich, UK; 4 Clinical Research Centre, Kingston General Hospital, Kingston, Ontario, Canada; 5 Division of Allergy and Immunology, Department of Medicine, Queen’s University, Doran 1, Kingston General Hospital, 76 Stuart Street, Kingston, ON K7L 2 V7, Canada

**Keywords:** Allergic rhinitis, Environmental exposure unit, Fexofenadine, PF-03654764, pseudoephedrine, H_3_ receptor, H_1_ receptor, Ragweed, Nasal congestion, Decongestant

## Abstract

**Background:**

Oral antihistamines that target the histamine receptor–1, such as fexofenadine, offer suboptimal relief of allergic rhinitis-associated nasal congestion. Combinations with oral sympathomimetics, such as pseudoephedrine, relieve congestion but produce side effects. Previous animal and human studies with histamine receptor-3 antagonists, such as PF-03654764, demonstrate promise.

**Methods:**

Herein we employ the Environmental Exposure Unit (EEU) to conduct the first randomized controlled trial of PF-03654764 in allergic rhinitis. 64 participants were randomized in a double-blind, placebo-controlled 4-period crossover study. Participants were exposed to ragweed pollen for 6 hours post-dose in the EEU. The primary objective was to compare the effect of PF-03654764 + fexofenadine to pseudoephedrine + fexofenadine on the subjective measures of congestion and Total Nasal Symptom Score (TNSS). The objectives of our post-hoc analyses were to compare all treatments to placebo and determine the onset of action (OA). This trial was registered at ClinicalTrials.gov (NCT01033396).

**Results:**

PF-03654764 + fexofenadine was not superior to pseudoephedrine + fexofenadine. In post-hoc analyses, PF-03654764 + fexofenadine significantly reduced TNSS, relative to placebo, and OA was 60 minutes. Pseudoephedrine + fexofenadine significantly reduced congestion and TNSS, relative to placebo, with OA of 60 and 30 minutes, respectively. Although this study was not powered for a statistical analysis of safety, it was noted that all PF-03654764-treated groups experienced an elevated incidence of adverse events.

**Conclusions:**

PF-03654764 + fexofenadine failed to provide superior relief of allergic rhinitis-associated nasal symptoms upon exposure to ragweed pollen compared to fexofenadine + pseudoephedrine. However, in post-hoc analyses, PF-03654764 + fexofenadine improved TNSS compared to placebo. Side effects in the PF-03654764-treated groups were clinically significant compared to the controls.

## Introduction

Allergic rhinitis is characterized by IgE and histamine-mediated symptoms such as rhinorrhea, sneezing, nasal pruritus, congestion, and aggravation of comorbid asthma [1]. Histamine interacts with 4 receptor subtypes, designated, H_1_, H_2_, H_3_ and H_4_. Antihistamines affecting the H_1_ receptor are widely used for acute relief of allergic rhinitis [2]. These agents, including fexofenadine, effectively reduce sneezing and rhinorrhea, but have limited effectiveness against congestion [3-6]. Congestion may be mediated by dilatation of venous capacitance vessels and extravascular plasma leak [7]. As vascular tone is under sympathetic neural control, traditional antihistamines have been combined with sympathomimetic decongestants such as pseudoephedrine [8,9]. However, due to side effects such as insomnia and hypertension, these agents are contraindicated in those with cardiovascular problems [8-11]. The development of H_3_ receptor antagonists as decongestants may represent a significant advance in available treatments.

Animal models demonstrated that H_3_ receptor antagonists inhibit nasal congestion in combination with antihistamines that target the H_1_ receptor [12-15]. Activation of the prejunctional histamine H_3_ receptors modulates sympathetic control of nasal vascular tone and resistance [15]. Oral administration of molecules that interfere with both the H_1_ and H_3_ receptors significantly attenuated total nasal symptoms, and nasal blockage, relative to placebo, but did not provide greater relief than cetirizine [16]. Thus, the present study was targeted towards examining a specific H_3_ receptor antagonist using an alternate active control, pseudoephedrine + fexofenadine, which is known to relieve congestion.

PF-03654764 is a potent and specific H_3_ receptor antagonist, with >1000-fold selectivity for the H_3_ receptor over the other histamine receptor subtypes [17]. A previous clinical trial employing a nasal ragweed bolus after administration of PF-03654746 (an H_3_ receptor antagonist with similar structure to PF-03654764) + fexofenadine demonstrated significantly reduced congestion, compared to placebo [11]. However, PF-03654764 has never been tested in a randomized controlled trial of allergic rhinitis, or in the Environmental Exposure Unit (EEU), which better approximates real life by delivering the allergen in the ambient air [18,19]. Therefore, our primary objective was to compare the effect of PF-03654764 + fexofenadine to pseudoephedrine + fexofenadine, on subjective measures of allergen-induced congestion and Total Nasal Symptom Score (TNSS) in the EEU, a sensitive, specific and reproducible methodology for allergen challenge. An exposure period of 6 hours post administration of study medication also enabled us to assess the onset of action.

## Methods

### Additional experimental details are available in an online data supplement

All study procedures were approved by the Queen's University Health Sciences and Affiliated Teaching Hospitals Human Research Ethics Board and all participants provided written informed consent before undergoing any study-specific procedures. This trial was registered at ClinicalTrials.gov (NCT01033396).

### Participant selection and enrollment

Exposure to ragweed pollen in the EEU is a well-tolerated, well-validated and reproducible method to elicit symptoms in a cohort of individuals with ragweed-induced allergic rhinitis [18]. Study participants were recruited from an existing database of potential research participants in the Allergy Research Unit, Kingston General Hospital, through posters placed throughout Kingston, Ontario, and the Queen’s University campus, as well as through local radio, and newspaper advertisements. The study was conducted out of ragweed season. Briefly, exclusion criteria included asthma requiring more than 3 uses per week of short acting inhaled β-agonists and severe hypertension. Blood pressure (BP) was measured at screening, pre and post-study period (pollen exposures), and follow-up.

### Dose selection

Doses of fexofenadine (60 mg) and pseudoephedrine (120 mg) were based on commercially available preparations. Previous drug-drug interaction (DDI) studies were not performed with PF-03654764. However, the similar H_3_ receptor antagonist, PF-03654746 was administered in combination with fexofenadine by Stokes et al. [11]. Pharmacokinetic drug interaction studies demonstrated low potential of PF-03654764 to inhibit activities of CYP 1A2, 2B6, 2C8, 2C9, 2C19, 2D6, and 3A4 based on IC50 values >30 μM versus the projected clinically efficacious concentration of 2.3 nM [20]. Nevertheless, participants were asked to abstain from grapefruit-related citrus fruits from 7 days prior to the first dose until collection of the final pharmacokinetic blood sample. The oral dose of 5 mg PF-03654764 was chosen to yield a mean maximum plasma concentration of 14 ng/mL, approximately 10 × Ki (10 times the binding affinity of PF-03654764 for the H_3_ receptor). This dose had previously been tested upon single and repeat dosing in healthy volunteers and had been generally safe and well tolerated [20]. Participants were instructed to abstain from all food and drink (except water) for 2 hours prior to dosing. Plasma clearance and half-life for PF-03654764 in humans were projected to be 3 mL/min/kg, and 16 hours, respectively [20].

### Priming visits

During a maximum of five priming visits, study participants were exposed to a target concentration of 3500 ± 500 grains/m^3^ of ragweed pollen (*Ambrosia artemisiifolia,* Greer Laboratories, Lenoir, North Carolina) for up to 3 hours, while recording their symptoms at baseline and every 30 minutes thereafter. Symptom score endpoints consisted of a four point (0 – 3) self-reported scale (definitions provided in Additional file 1: Table S4), for each of the following symptoms: congestion, sneezing, nasal itch, rhinorrhea. Total nasal symptom score (TNSS) was the sum of the scores for sneezing, nasal itch and rhinorrhea (maximum score of 9). Participants who did not achieve a TNSS equal to or greater than 4 and a congestion score equal to or greater than 2 at the 90-minute time point during at least one priming visit were excluded from the study to ensure medication effects would be discernible.

### Study periods

The first study period took place within 12 days of priming and periods were separated by 2-weeks. Participants were exposed to a target concentration of 3500 ± 500 grains/m^3^ of ragweed pollen for eight hours (-2H to 6H, with administration of medication at 0H). Participants were not randomized until rhinitis symptoms were verified just prior to dosing (TNSS ≥ 4 and a congestion score ≥ 2 at the 90-minute time point). To maintain participant and investigator blinding, three pills, identical in appearance, were dispensed from three pairs of bottles (active vs placebo for each of three active treatments). Participants were allocated to a treatment sequence using a computer-generated randomization schedule (Table 1). The allotment of participants to different treatment sequences was not equal in order to give more power to the primary comparison (fexofenadine + pseudoephedrine *vs*. fexofenadine + PF-03654764).

**Table 1:** Sequences of study treatment administrations

**Sequence**	**Number of participants**	**Study period**			
		**1**	**2**	**3**	**4**
**1**	4	B	D	B	C
**2**	4	B	B	C	D
**3**	4	C	B	D	B
**4**	4	D	C	B	B
**5**	12	A	C	A	C
**6**	12	C	A	C	A
**7**	12	A	C	C	A
**8**	12	C	A	A	C

A: PF-03654764 capsule (5 mg) + fexofenadine capsule (60 mg) + placebo capsule.

B: PF-03654764 capsule (5 mg) + placebo capsule + placebo capsule.

C: placebo capsule + placebo capsule + fexofenadine (60 mg)/pseudoephedrine (120 mg) capsule.

D: placebo capsule + placebo capsule + placebo capsule.

### Statistics

The sample size was calculated based on mean score from hour 2 to hour 6 for congestion and TNSS, and a Bayesian interpretation of the results, assuming a non-informative prior and a within-subject standard deviation of 0.7. The primary efficacy analyses used sequences 5–8 and included all participants randomized who received at least one dose and who had at least one post-dose measurement. A mixed effect model was used, with participant as a random effect, period/treatment as fixed effects and baseline covariates. Baseline was calculated as the mean of the last two pre-treatment symptom scores (-0.5H and 0H). The baseline covariates were:

Baseline mean = mean pre-dose value for each participant across the four periods

Baseline difference = the difference between the pre-dose value at a given period and the participant’s baseline mean

Differences between treatment means, standard errors (SE), and two-sided 90% confidence intervals (CI) are presented.

In general, Studentized residuals are preferable to standardized residuals for purposes of outlier identification, and values of 3 or greater (or -3 or less) may be considered outliers [21]. Therefore, to avoid the situation where an extreme data point can be highly influential in the analysis, data points that met these criteria were excluded, and when this occurred, all data from the participant was removed. These exclusion criteria were decided upon *a priori* in advance of study commencement.

The criteria used for evaluation of the efficacy and non-inferiority of PF-03654764 + fexofenadine against congestion compared to existing treatment were:

Criteria 1: At least 80% sure that PF-03654764 + fexofenadine has a greater than 0.15 point reduction in congestion compared to pseudoephedrine + fexofenadine (for efficacy).

Criteria 2: At least 80% sure that PF-03654764 + fexofenadine was non-inferior to pseudoephedrine + fexofenadine, using a non-inferiority margin of -1.

### Post-Hoc analyses

The investigator’s site undertook post-hoc analyses to compare all groups to placebo. Mean symptom scores were calculated by taking the least-squares mean between 2H and 6H. Methods above were used for the post-hoc ANCOVA, except all groups were included to compare to placebo. Onset of action (OA) was calculated for treatments found to significantly relieve symptoms, defined as the median onset of a clinically important reduction (0.5 symptom score units from baseline for congestion; 1.0 for TNSS) across all participant study periods. All post-hoc analyses were conducted under the auspices of the investigator site. Graphing was performed using Prism Version 4.0c (GraphPad Inc., La Jolla, CA). All other statistical analyses were carried out using SAS Version 9.3 (SAS Institute Inc., Cary, NC).

## Results

Demographic data on the participants in this study are presented in Table 2. All participants had a minimum 2 year history of allergic rhinitis to ragweed. The participants self-reported being diagnosed with allergic rhinitis by a physician between 6 and 56 years prior to enrolment in the study (mean duration 25.3 years). The average pollen concentrations determined *via* Rotorod^®^ samplers in the EEU were 2913 ± 216 grains/m^3^ for priming visits and 3348 ± 103 grains/m^3^ for treatment visits.

**Table 2:** Participant demographic data

**Characteristic**	**Total (n = 64)**
Age (years)	39.7 (19–59)
Female Sex (%)	51.6%
Caucasian (%)	90.6%
Body Mass Index (kg/m^2^)	26.8 (19.7 – 32.0)

Demographic data for participants in this study. Mean age in years (range). Body Mass Index in kg/m^2^ (range).

### Symptom time-course

**Figure 1:**
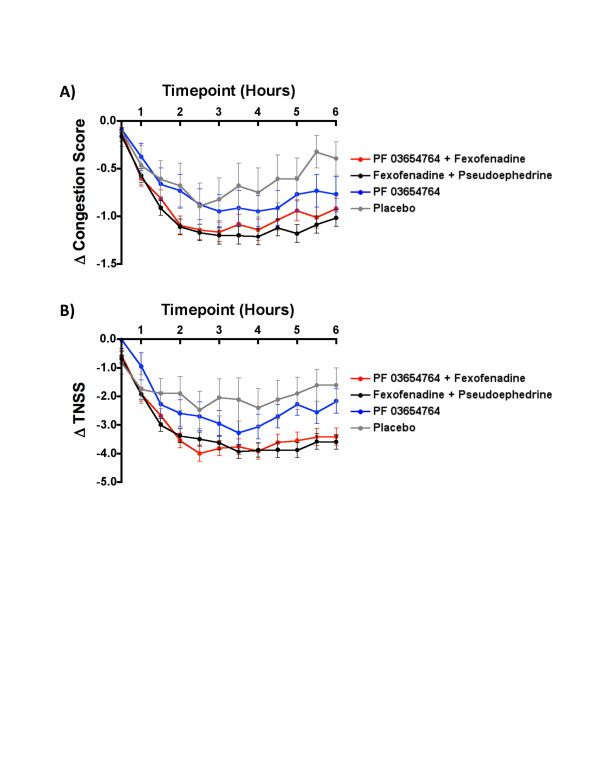
**Time-course of change in symptom scores post-treatment.** Exploratory visualization of the change in symptom scores (Δ to baseline) are shown for **A)** Congestion, and **B)** Total Nasal Symptom Score (TNSS) from time of drug administration (0 hours) to 6 hours post-treatment. Lines and error bars represent the raw means and standard errors of the means for each of the treatments over time. It includes all participant study periods in which a participant was administered a treatment and remained in the EEU for the entire study period (excludes those periods where a participant discontinued the study without completing the visit). The y-intercept represents baseline, defined as the mean of the last two pre-treatment symptom scores (-0.5H and 0H). Samples sizes were; n = 86, 96, 28 and 14 for PF-03654764 + fexofenadine, fexofenadine + pseudoephedrine, PF-03654764, and placebo, respectively.

During each study period, participants developed ragweed-induced symptoms of allergic rhinitis during the 2 hours prior to treatment (-2H to 0H). The effectiveness of the different treatment arms against congestion and total nasal symptom scores (TNSS) over time can be visualized as the post-treatment change from baseline (Figure 1).

### Primary efficacy analysis

The primary efficacy analysis employed treatment sequences 5–8 to compare PF-03654764 + fexofenadine to fexofenadine + pseudoephedrine. Six and nine participant-treatment periods were lost due to discontinuations, illnesses and contraindicated medication use in the PF-03654764 + fexofenadine and fexofenadine + pseudoephedrine groups, respectively. The exclusion of two outliers further reduced the PF-03654764 + fexofenadine group by four participant treatment periods. Thus, Table 3 presents the primary efficacy analyses derived from a total of 86 and 87 participant treatment periods, in the PF-03654764 + fexofenadine and fexofenadine + pseudoephedrine groups, respectively. PF-03654764 + fexofenadine was not effective in reducing nasal congestion compared to pseudoephedrine + fexofenadine (i.e., Criteria 1 was not met). However, PF-03654764 + fexofenadine was shown to be non-inferior to pseudoephedrine + fexofenadine with respect to TNSS (i.e., Criteria 2 was met).

**Table 3:** Mixed model ANCOVA of mean congestion and TNSS scores (2–6 hours)

				**Difference (test – reference)**			
**Sequences 5–8: congestion**							
	**N**	**Adjusted mean**	**SE**	**Adjusted mean**	**SE**	**90% CI**	**Prob** **<-0.15**
**PF-03654764**	86	1.58	0.106	0.07	0.080	(-0.06, 0.20)	0.0035
**+ fexofenadine**							
**Pseudoephedrine**	87	1.51	0.107				
**+ fexofenadine**							
**Sequences 5–8: TNSS**							
	**N**	**Adjusted Mean**	**SE**	**Adjusted Mean**	**SE**	**90% CI**	**Prob NI**
**PF-03654764**	86	3.81	0.288	-0.08	0.233	(-0.46, 0.31)	1.000
**+ fexofenadine**							
**Pseudoephedrine**	87	3.89	0.289				
**+ fexofenadine**							

The primary analysis consisted of a mixed effects ANCOVA with period and treatment fixed effects and subject random effect, employing sequences 5–8 to compare PF-03654764.

+ fexofenadine to pseudoephedrine + fexofenadine. N = number of non-missing observations included in the analysis.

### Post-Hoc efficacy analysis

The investigator’s site undertook a post-hoc analysis, that was not part of the pre-specified statistical plan, to compare the treatments of interest to placebo. The sample size difference in the fexofenadine + pseudoephedrine group reflects the addition of participants from sequences 1–4. In the placebo group 16 participants were assigned to treatment but one never received placebo, as they were assigned to sequence 2 and dropped out of the study after study period 2. In the PF-03654764 group 16 participants were randomized but one discontinued during period 2 in sequence 4 and thus never received PF-03654764 during the following two periods (3 and 4). One participant in sequences 1–4 was excluded as an outlier according to the pre-specified exclusion criteria, reducing the sample sizes for placebo and PF-03654764 to 14 and 28, respectively. Pseudoephedrine + fexofenadine significantly reduced congestion and TNSS, compared to placebo (Table 4). PF-03654764 alone did not have a significant effect on symptoms. In the post hoc mixed model, the combination of PF-03654764 with fexofenadine significantly improved TNSS, but not congestion (Table 4).

**Table 4:** Post-Hoc analysis of mean symptom scores (2H – 6H)

**Treatment**	**N**	**Parameter**	**Mean ± SE**	**95% Confidence interval**	**Adj. p-value to placebo**
**Placebo**	14	Congestion	2.00 ± 0.18	(1.64, 2.36)	N/A
		TNSS	5.51 ± 0.50	(4.53, 6.49)	N/A
**PF-03654764**	28	Congestion	1.90 ± 0.15	(1.59, 2.20)	0.939
		TNSS	4.84 ± 0.42	(4.00, 5.67)	0.186
**PF-03654764 + fexofenadine**	86	Congestion	1.58 ± 0.10	(1.39, 1.78)	0.138
		TNSS	3.63 ± 0.27	(3.10, 4.16)	0.001
**Pseudoephedrine + fexofenadine**	97	Congestion	1.50 ± 0.09	(1.31, 1.68)	0.034
		TNSS	3.70 ± 0.25	(3.20, 4.20)	0.003

Mean symptom scores for congestion and Total Nasal Symptom Score (TNSS) were calculated by taking the least squares mean between 2H and 6H post-treatment. Standard error (SE) of the mean and 95% confidence intervals were also calculated for each treatment. N = number of participant study periods on each treatment. Groups were compared using a mixed model ANCOVA and the Tukey-Kramer adjustment was used to control for multiple comparison.

### Onset of action

The median onset of action for pseudoephedrine + fexofenadine regarding congestion and TNSS was 60 minutes and 30 minutes, respectively. The median onset of action for PF-03654764 + fexofenadine against TNSS was 60 minutes.

### Adverse events

There were no serious adverse events (SAEs). Systolic BP increases from baseline ≥30 mm Hg were experienced by 2/61 and 1/15 participants receiving fexofenadine + pseudoephedrine and placebo, respectively. Increases in diastolic BP ≥30 mm Hg were experienced by 1/47, 4/61, 1/15 participants receiving PF-03654764 + fexofenadine, fexofenadine + pseudoephedrine and placebo, respectively. Out of the 64 participants who were randomized, four, two, one, and zero discontinued during treatment with PF-03654764 + fexofenadine, pseudoephedrine + fexofenadine, PF-03654764, and placebo, respectively. Two participants, both receiving PF-03654764 + fexofenadine, discontinued due to AEs that were considered to be treatment-related. As mentioned above, one participant never received placebo and one never received PF-03654764, thus sample sizes for both of those groups are fifteen participants for AE analysis. Three participants slated to receive fexofenadine + pseudoephedrine were never treated due to discontinuations in previous study sessions. The incidence of AEs, both all causality and treatment-related, was greater in the PF-03654764-alone and PF-03654764 + fexofenadine groups, compared to the placebo and pseudoephedrine + fexofenadine groups (Table 5). The most common treatment-related AEs were insomnia, headache and nausea (Table 6).

**Table 5:** Summary of treatment-emergent adverse events by participant

**Adverse Event (AE)**	**PF-03654764 + fexofenadine (N = 48)**	**PF-03654764 (N = 15)**	**Pseudoephedrine + fexofenadine (N = 61)**	**Placebo (n = 15)**
**All Causalities**				
Number of AEs	116	39	30	3
Participants with AEs	36 (75)	12 (80)	18 (29.5)	3 (20)
Participants with severe AEs	23 (49)	6 (40)	4 (6.6)	0
Participants discontinued due to AEs	2 (4.2)	1 (6.7)	0	0
**Treatment-Related**				
Number of AEs	83	30	11	0
Participants with AEs	22 (45.8)	11 (73.3)	6 (9.8)	0
Participants with severe AEs	17 (35.4)	6 (40.0)	2 (3.3)	0
Participants discontinued due to AEs	2 (4.2)	0	0	0

Summary of AEs by participant across all dosing periods. N values represent the number of participants randomized to a sequence receiving one or more administrations of the treatment that were actually administered the treatment. In brackets, the percentage of participants experiencing adverse events with that treatment is given. Note that participants had twice as many opportunities to experience AEs on a particular treatment if they received the same treatment during two separate dosing periods.

**Table 6:** Incidence of most frequent treatment-related adverse events (≥5% per treatment, by participant)

**Adverse Event (AE)**	**PF-03654764 + fexofenadine (N = 48)**	**PF-03654764 (N = 15)**	**Pseudoephedrine + fexofenadine (N = 61)**	**Placebo ** **(N = 15)**
**Insomnia**	10 (20.8)	5 (33.3)	3 (4.9)	0
**Headache**	9 (18.8)	3 (20.0)	1 (1.6)	0
**Nausea**	9 (18.8)	4 (26.7)	1 (1.6)	0
**Dysgeusia**	5 (10.4)	0	1 (1.6)	0
**Hot flush**	4 (8.3)	1 (6.7)	1 (1.6)	0
**Nightmare**	4 (8.3)	0	0	0
**Abnormal Dreams**	3 (6.3)	0	0	0
**Dizziness**	3 (6.3)	1 (6.7)	2 (3.3)	0
**Malaise**	3 (6.3)	0	0	0
**Night Sweats**	3 (6.3)	3 (20.0)	0	0
**Tachycardia**	3 (6.3)	0	0	0
**Diarrhea**	2 (4.2)	1 (6.7)	0	0
**Palpitations**	2 (4.2)	1 (6.7)	1 (1.6)	0
**Fatigue**	1 (2.1)	1 (6.7)	0	0
**Hallucination**	1 (2.1)	2 (13.3)	0	0
**Upper abdominal pain**	0	1 (6.7)	0	0
**Feeling Jittery**	0	1 (6.7)	0	0
**Feeling of body temperature change**	0	1 (6.7)	0	0
**Cognitive disorder**	0	1 (6.7)	0	0
**Migraine**	0	1 (6.7)	0	0
**Parosmia**	0	1 (6.7)	0	0
**Anxiety**	0	1 (6.7)	0	0
**Hyperhidrosis**	0	1 (6.7)	0	0

N = number of participants (percentage of total receiving that treatment). AEs were ranked in order of decreasing frequency by PF-03654764 + fexofenadine (treatment of interest).

## Discussion

In this study we examined the combination of the H_3_ receptor antagonist, PF-03654764, and the H_1_ receptor antihistamine, fexofenadine, for the first time as a potential treatment for allergic rhinitis. This investigational treatment was not superior to established treatment. In post-hoc analyses we demonstrated that PF-03654764 + fexofenadine significantly reduced Total Nasal Symptom Score (TNSS), relative to placebo. Although this study was not powered to conduct statistical evaluations of safety, the incidence of adverse events (AEs) was greater in PF-03654764-treated groups, compared to existing treatment or placebo.

### Nasal congestion

The primary objective of this study was to compare the effect of PF-03654764 + fexofenadine on allergic rhinitis-induced nasal congestion to a commercially available dose of fexofenadine + pseudoephedrine. Neither PF-03654764 nor the PF-03654764 + fexofenadine combination was effective at reducing congestion. Additionally, these treatments did not meet the decision criteria for superiority over existing therapy. While PF-03654764 was not significantly better than placebo, the combination with fexofenadine was significantly better than PF-03654764 by itself on TNSS, and the combination of PF-03654764 with fexofenadine was significantly better than placebo on TNSS. This posits the question of whether the results are due to the fexofenadine and not PF-03654764. Dose adjustment studies would be able to address this issue and determine if changes in oral dose of PF-03654764 affect the outcome of the experiment. In Stokes et al. there was a clear dose-dependent phenomenon with a very similar H_3_ receptor antagonist, and it would be interesting to see if a similar pattern exists with PF-03654764.

### H_3_ receptor antagonists and nasal congestion

A previous clinical study investigated dual H_1_/H_3_ receptor interference in an environmental challenge chamber [16]. Both nasal blockage and TNSS were improved, compared to placebo, but the treatment similarly failed to prove superior to existing treatment, in that case, cetirizine, an H_1_ receptor antihistamine [16]. As that study employed a non-specific molecule, and an active control that is not effective against congestion, this study was targeted towards examining a specific H_3_ receptor antagonist using an alternate active control, pseudoephedrine + fexofenadine.

Recently, Stokes *et al.* employed acoustic rhinometry to measure changes in minimum nasal cross-sectional area and nasal volume after treatment with PF-03654746 + fexofenadine (an H_3_ receptor antagonist with a similar structure to PF-03654764) [11]. The treatment significantly reduced subjective congestion scores, compared to placebo, but improvements in rhinometry measurements were not detected [11]. Those results may be explained in view of the disconnect between perception of nasal airflow and congestion and physical characteristics of intranasal space. However, since our primary objective was to see the efficacy on congestion, the non-significant response to H_3_ antagonism to measured vascular congestion in humans is important. As 5 mg of PF-03654764 yields a plasma concentration of approximately 10 × Ki (10 times the binding affinity of PF-03654764 for the H_3_ receptor), effective H_3_ receptor antagonism should have been achieved in our study. However, Stokes and colleagues employed a similar but distinct H_3_ antagonist to the one used herein, with a relatively higher dose (up to 30 × Ki) [11].

### Experimental allergen challenge methods

Major differences between our protocol and Stokes *et al.* may also drive some of the differences in our findings. Timing of symptom development in relation to drug administration and the allergen challenge method employed are key. Stokes *et al.* investigated prophylactic potential, performing the nasal allergen provocation post-drug administration at the time that the drug serum concentration was estimated to be maximal [11]. Ours is the first study to examine the potential for specific H_3_ receptor antagonists to *relieve* pre-existing allergy symptoms induced by inhalation of allergen in the ambient air. We administered the study drug after 2 hours of allergen exposure, followed by an additional 6 hours of post-drug allergen exposure and symptom recording and were able to report the onset of action against allergic rhinitis symptoms for the first time. The advantages and disadvantages of different methods of allergen challenge for evaluating novel therapeutic preparations have been discussed elsewhere [18,22]. In brief, nasal allergen provocation tests have the advantage of focusing on a smaller number of participants at a time, enabling the collection of data such as acoustic rhinometry. However, as Stokes *et al.* reported no significant effect on minimum nasal cross-sectional area or nasal volume [11], the present study did not evaluate that outcome. Instead, we employed the EEU to allow larger numbers of study volunteers to be simultaneously exposed to standardized ambient levels of ragweed pollen, providing the recognized advantage of a more natural mode of allergen exposure [18]. Ragweed allergen was chosen, as it is one of the most common aeroallergens that naturally induces allergic rhinitis symptoms in our study population, and the level of exposure in the EEU was consistent with peak seasonal levels [23,24]. European annual mean pollen counts of ragweed are as high as 7800 grains/m^3^[3,25]. Although ragweed pollen is not as common in the United States, weed pollens do reach as high as 2900 grains/m^3^[3,26] and tree pollen is known to reach over 8000 grains/m^3^[3,27]. It was recently demonstrated that there is a high degree of concordance between allergic symptoms induced on exposure to pollen in an environmental exposure unit and those experienced during the natural season [28]. An outpatient study during ragweed season may have achieved similar efficacy results, but could not have been used to evaluate the onset of action [18].

### Onset of action

The time to onset of action for PF-03654764 + fexofenadine has not previously been reported. In this study we report that it is 60 minutes for clinically important improvement in TNSS. For fexofenadine + pseudoephedrine we determined a 60 minute onset of action against congestion, and a 30 minute onset of action against TNSS. We previously found that fexofenadine alone provided clinically important relief from allergic rhinitis symptoms at 60 minutes [29]. The onset of action for fexofenadine + pseudoephedrine has been previously reported by Berkowitz *et al.* to be 45 minutes [30]. Differences in reported onset of action likely result from timing of diary card collection and the symptoms included in composite scores. Overall, the onset of action is remarkably consistent between environmental exposure unit studies, and the slower onset of action against congestion compared to TNSS may reflect a lag in the subjective perception of nasal fullness compared to the decline in number of sneezes and other symptoms.

### Adverse events

In this study PF-03654764-treated groups exhibited a higher incidence of AEs, compared to pseudoephedrine + fexofenadine or placebo. Previously, Stokes *et al.* reported greater AEs in participants treated with the similar H_3_ receptor antagonist, PF-03654746 [11]. Many of the AEs in this study are consistent with those reported by Stokes *et al.*, including insomnia, hallucination and feeling jittery [11]. H_3_ receptors have been demonstrated in the central nervous system, where they regulate other neurotransmitters (e.g. acetylcholine and norepinephrine) [17,31]. Additionally, PF-03654764 is believed to be fully central nervous system penetrant, which may also help to explain the finding of increased AEs and their nature. It was envisioned that the combination of a novel H_3_ receptor antagonist with an established antihistamine may provide relief with a superior safety profile for those who are contraindicated to treatment with sympathomimetic agents [11,17]. Another H_3_ receptor antagonist with a lesser penetrance into the central nervous system may exhibit a more favorable safety profile, or even increased efficacy against allergic rhinitis symptoms.

### Limitations

A limitation of this study is that we are unable to make a direct comparison between fexofenadine and the fexofenadine + PF-03654764 combination. A fexofenadine monotherapy treatment was not included in this study, as the primary goal of the trial was to assess the effects of fexofenadine + PF-03654764 vs. the existing combination therapy of fexofenadine + pseudoephedrine. In future studies it would be interesting to include a fexofenadine monotherapy group so that the effect difference could be compared to H_3_ antagonist monotherapy to assess synergistic/antagonistic effects.

Also of note, it has recently been demonstrated that common antihistamines may not act as simple antagonists, but rather as inverse agonists, stabilizing inactive forms of the H_1_ and H_2_ receptors [32-34]. Inverse agonism describes the ability of certain “antagonists” to reduce the activity of receptor systems that are active in the absence of agonists [35]. However, whether inverse agonism is essential or clinically important for antihistamines has not been clarified yet [35]. Thus far, the published literature indicates that PF-03654764 is a potent and specific H_3_ receptor antagonist [17]. However, other H_3_ receptor “antagonists” have recently been shown to exhibit inverse agonist activity [36]. Further biochemical and pharmacological studies are needed to understand the mechanism of action in detail.

## Conclusions

In conclusion, we demonstrated that the H_3_ receptor antagonist, PF-03654764, plus the H_1_ receptor antagonist, fexofenadine, did not achieve superiority over established treatment in a double-blind, placebo-controlled crossover study in the Environmental Exposure Unit. However, we demonstrated non-inferiority compared to pseudoephedrine + fexofenadine.

## Abbreviations

AE: Adverse event; ANCOVA: Analysis of covariance; BP: Blood pressure; CI: Confidence interval; EEU: Environmental exposure unit; Ki: Binding affinity; MMRM: Mixed model repeated measures; OA: Onset of action; PF-03654764: Trans-N-ethyl-3-fluoro-3-[3-fluoro-4-(pyrrolidinylmethyl) phenyl] cyclobutanecarboxamide; SAE: Serious adverse event; SE: Standard error; TNSS: Total nasal symptom score.

## Competing interest

The authors declare that they have no conflicts of interest. RJA and FH were employees of Pfizer Ltd., United Kingdom, at the time of study conduct.

## Authors’ contributions

MLN carried out data analysis and interpretation, graphing and preparing figures, drafting the article. TW was involved in the conception and design of the study and carried out the collection of data with regards to pollen exposure and pollen levels during the challenges, analysis and interpretation of pollen data. LMS was involved in the conception and design of the study with regards to ethics, measures and endpoints, and carried out data collection. BGH was involved in the conception and design of the study with regards to logging symptom score data, data collection, security and export for statistical analysis. RJA was involved in the conception and design of the study with regards to study drug and major endpoints and carried out interpretation of data. FH was involved in the conception and design of the study with regards to statistics, and carried out data interpretation and statistical analysis of the primary outcomes. XS carried out post-hoc statistical analysis. AGD was involved in the conception and design of the study with regards to post-hoc statistical analysis, and carried out interpretation of data. AKE was involved in the conception and design of the study with regards to pollen exposure, clinical outcomes, symptom scores, and carried out the collection and interpretation of data. All Authors contributed to revising the manuscript for important intellectual content and gave their final approval of the version to be published.

## Supplementary Material

Additional file 1 Supplementary Methods. Click here for file
