# Supplementary material for: Add-on histamine receptor-3 antagonist for allergic rhinitis: a double blind randomized crossover trial using the environmental exposure unit
Source: Allergy Asthma Clin Immunol. 2014 Jul 3;10(1):33. doi: 10.1186/1710-1492-10-33 (PMC4094756; doi:10.1186/1710-1492-10-33)
Supplement: Additional file 1 — Supplementary Methods. [file 1710-1492-10-33-S1.doc]

**Add-on histamine receptor-3 antagonist for allergic rhinitis: A double blind randomized crossover trial using environmental exposure unit: Online Supplement**

Michelle L. North PhD^1,2^, Terry Walker BA^2^, Lisa M. Steacy BSc CCRP^2^, Barnaby G. Hobsbawn^2^, Richard J. Allan BSc^3^, Frances Hackman BSc^3^, Xiaoqun Sun MSc^4^, Andrew G. Day, MSc^4^ and Anne K. Ellis MD MSc^1,2,5^

^1^Department of Biomedical and Molecular Sciences, Queen’s University, Kingston, Ontario, Canada.

^2^Allergy Research Unit, Kingston General Hospital, Kingston, Ontario, Canada.

^3^Pfizer Ltd., United Kingdom.

^4^Clinical Research Centre, Kingston General Hospital, Kingston, Ontario, Canada.

^5^Division of Allergy and Immunology, Department of Medicine, Queen’s University, Kingston, Ontario, Canada.

**Inclusion Criteria**

Participants must have met all of the following inclusion criteria to be eligible for enrollment in the study:

1. Male or female participants aged 18-60 years. Female participants may be of child-bearing or non-child-bearing potential. Females of child-bearing potential must use appropriate birth control throughout the study and pregnancy tests must be negative prior to dosing at each study visit.
2. Participants with a positive skin prick test to short ragweed extract (defined as a wheal diameter ≥3 mm in excess of the wheal diameter of the diluent).
3. Participants with a TNSS of ≥4 and a congestion score ≥2 at the 90 minute time point during at least one priming session.
4. Participants with a TNSS of ≥4 and a congestion score ≥2 at the 90 minute time point during study period 1 prior to study medication administration.
5. Participants with a history of seasonal allergic rhinitis during the preceding 2 ragweed allergen seasons.
6. Body Mass Index (BMI) of 17.5 to 32 kg/m^2^; and a total body weight >50 kg (110 lbs).
7. An informed consent document signed and dated by the participant.
8. Participants who are willing and able to comply with the scheduled visits, treatment plan, laboratory tests and other study procedures.

**Exclusion Criteria**

Participants presenting with any of the following were not be included in the study:

1. Participants with evidence (on review of pre-study laboratory data and full physical examination) or history of clinically significant hematological, renal, endocrine, gastrointestinal, hepatic, psychiatric, neurological diseases other than changes related to allergic rhinitis. Participants with some stable chronic medical conditions not expected to interfere with the conduct or safety of the study were eligible to participate; these include controlled hypertension, controlled hypercholesterolemia, controlled diabetes and mild controlled osteoarthritis.
2. Participants with a laboratory abnormality that may increase the risk associated with study participation or investigational product administration or may interfere with the interpretation of study results and, in the judgment of the investigator, would make the participant inappropriate for entry into this study.
3. Participants with a known history of positive test results for Hepatitis, HIV or tuberculosis other than would be anticipated following vaccination.
4. Participants with a significant history of asthma (requiring more than 3 uses per week of short acting inhaled β–agonists) or chronic uticaria.
5. Participants with known non-responsiveness to anti-histamine treatment.
6. Participants who have suffered an upper or lower respiratory tract infection in the 2 weeks prior to screening.
7. Participants who have undergone a nasal or lung provocation procedure in the 4 weeks prior to screening.
8. Participants who have had clinically significant symptoms of allergic rhinitis (as judged by the investigator) within the 2 weeks before screening.
9. Participants with significant nasal deformity (greater than 50% obstruction) that could impact the results of the study as determined my the investigator or recent nasal surgery.
10. Participants with a history of nasal polyps or chronic rhinosinusitis that could impact the results of the study as determined by the investigator within the last 6 months.
11. Participants who are taking or have taken restricted concomitant medications (see Supplemental Table I, below).
12. Participants with known hypersensitivity to or intolerance of allergen challenge, fexofenadine or pseudoephedrine.
13. Participants with a history of hemorrhagic disorders, cerebrovascular accident or bleeding diatheses.
14. Participants with a history or presence of hypertension (systolic >140 mm Hg and/or diastolic >90 mm Hg) or significant cardiovascular disease.
15. Participants with a 12-lead ECG demonstrating QTc >450 ms at screening.
16. Participants with a clinically significant abnormal ECG at screening, particularly evidence of ischemic heart disease.
17. Participants with a history of prostatic hypertrophy or urinary retention, uncontrolled hyperthyroidism, uncontrolled diabetes mellitus or increased intraocular pressure.
18. Participants with a history of sensitivity to heparin or heparin-induced thrombocytopenia.
19. Participants who have evidence of alcohol or drug abuse.
20. Blood donation of approximately 1 pint (500ml) within 4 weeks prior to dosing.
21. In the opinion of the investigator a participant who is unlikely to complete the study for any reason.
22. Participants who have received treatment with an investigational drug within 28 days (or five half lives, whichever is longest) prior to screening.
23. Participants that have received a potent CYP3A4 inhibitor or inducer within 14 days of dosing of study medication.
24. Pregnant or nursing females; females of childbearing potential who are unwilling or unable to use acceptable birth control, prior to screening until completion of follow-up procedures.
25. Unwilling or unable to comply with the lifestyle guidelines, including;
    1. Abstaining from food and drink (except water) for at least 4 hours prior to safety laboratory evaluations and 2 hours prior to dosing
    2. Refraining from grapefruit or grapefruit related food/drink from 7 days prior to the first dose of study medication until the collection of the final pharmacokinetic blood sample,
    3. Abstaining from alcohol for 24 hours prior to admission to the Environmental Exposure Unit until discharge from each study period.
    4. Abstaining from the use of tobacco or nicotine-containing products prior to dosing and during confinement in the research unit.
    5. Abstaining from strenuous exercise for at least 48 hours prior to each blood collection for safety laboratory evaluations.
26. Participant is the investigator, sub-investigator, research assistant, pharmacist, study coordinator, other staff or a relative of study personnel directly involved in the conduct of the study.

Any other severe acute or chronic medical or psychiatric condition or laboratory abnormality that may increase the risk associated with study participation or investigational product administration or may interfere with the interpretation of study results.

***Serious Adverse Events***

A serious adverse event is defined as any untoward medical occurrence that meets any of the following criteria:

1. Results in death
2. Is life-threatening
3. Requires inpatient hospitalization
4. Results in persistent or significant disability
5. Results in congenital anomaly/birth defect

***Severity Assessment for Adverse Events***

When an adverse event was reported by a participant (ex., a headache) they were asked to describe the severity of the adverse event using the rating system in **Supplemental Table III**.

***Causality Assessment***

All adverse events were assessed by a medical doctor who determined whether there existed a reasonable possibility that the investigational product caused or contributed to the adverse event.

***Ragweed Allergen Exposures***

Prior to use, short ragweed pollen (*Ambrosia artemisiifolia,* Greer Laboratories, Lenoir, North Carolina) was independently tested for fungal and bacterial contamination (Paracel Laboratories, Ottawa, ON, Canada). To ensure consistency between sessions and uniform pollen distribution, pollen counts were performed from seven Rotorod® samplers (Sampling Technologies Inc, Minnetonka, Minnesota), distributed throughout the participant seating area every 30 minutes during all exposures ^1, 2^. Environmental variables including air quality, temperature, humidity, and carbon dioxide levels, were tightly regulated as described previously ^1, 2^. All data relating to pollen counts and air quality were stored and analyzed at the investigator’s site.

**Supplemental References**

1. Ellis AK, Ratz JD, Day AG, Day JH. Factors that affect the allergic rhinitis response to ragweed allergen exposure. Ann Allergy Asthma Immunol 2010; 104:293-8.

2. Day JH, Briscoe MP. Environmental exposure unit: a system to test anti-allergic treatment. Ann Allergy Asthma Immunol 1999; 83:83-9; quiz 9-93.

**Supplemental Table I: Medications and Substances Contraindicated throughout the Study**

| **Medication or Substance** | **Washout Period** |
| --- | --- |
| 1) Grapefruit or related citrus juice or fruit | 7 days |
| 2) Intra-nasal, ocular and systemic decongestants (including dietary supplements containing ephedrine) | 72 Hours |
| 3) Long Acting β Agonists  4) Tricyclic antidepressants and mono-amine oxidase inhibitors  5) Potent CYP3A4 inhibitors or inducers  6) Intra-nasal or inhaled anti-muscarinic agents | 14 days |
| 7) Corticosteroids, of all forms except topical hydrocortisone ≤1% covering ≤10% of the body surface area | 28 days |
| 8) Investigational drugs | 28 days or 5 half lives, whichever is longest |
| 9) Omalizumab  10) Immunotherapy injections not in maintenance phase | 6 months |

Medications and substances contraindicated in the time period stated before priming visits and for the duration of the study.

**Supplemental Table II: Medications and Substances Contraindicated Prior to Each Pollen Exposure**

| **Medication or Substance** | **Washout Period** |
| --- | --- |
| 1) Immunotherapy injections (maintenance phase) | 48 hours |
| 2) Antihistamines (oral, ocular, intra-nasal)  3) Leukotriene receptor antagonists  4) Theophylline  5) Non-steroidal anti-inflammatory agents  6) Systemic antibiotics  7) Intra-nasal and ocular cromolyns | 7 days |

Medications and substances contraindicated in the time period prior to each pollen exposure.

**Supplemental Table III:** Severity Assessment for Adverse Events

| **Classification** | **Description** |
| --- | --- |
| Mild | Does not interfere with participant’s usual function. |
| Moderate | Interferes to some extent with participant’s usual function. |
| Severe | Interferes significantly with participant’s usual function. |

Rating system to describe the severity of adverse events.

**Supplemental Table IV: Symptom Scoring System**

| **Score** | **Classification** | **Description** |
| --- | --- | --- |
| 0 | None | Symptom is completely absent. |
| 1 | Mild | Symptom is present, but not bothersome. |
| 2 | Moderate | Symptom is bothersome, but tolerable. |
| 3 | Severe | Symptom is hard to tolerate, desiring treatment. |

Rating system to describe the severity of symptoms. Participants were asked to record symptom scores on diary cards every 30 minutes for the duration of the study to assess congestion, sneezing, nasal itch, and rhinorrhea.
